# Supplementary material for: Chromothripsis during telomere crisis is independent of NHEJ, and consistent with a replicative origin
Source: Genome Res. 2019 May;29(5):737–49. doi: 10.1101/gr.240705.118 (PMC6499312; doi:10.1101/gr.240705.118)
Supplement: Supplemental Material [file supp_gr.240705.118_Supplemental_file_1.zip › contigs/annotated_contigs/DB39/contig.3.DB39_length_546_mean_cov_7.79304029304.docx]

**DB39_length_546_mean_cov_7.79304029304**

```
CAAACC|CACAAATAAAAAAAAAAAAAAAAAAAAAAAAAAAGAAAAAAAAAAAAAAAAAA|TAAAAAAAAAAAAAAACAAAAAAAATTA
       >chr5:156284108-156284161 - E=3e-14 p=0e+00           >chr12:126975637-126975675
AAAAGAAGAG|G|TTTTTTTTTTTTTTTTTTTTTGAGACAGAGTCTCACTCTGTTGCCCAGGCTGGAGTACAATGGCACAATCTCGGCT
 + E=3e-05  >chr3:186480690-186481120 + E=1e-190
CACTGCAACCTCTGCCTCCCGGGTTCAAGCAATTATCTTGCTCAGCTTCTTGAGTAGCTGGAATTATAGGCACATGCCACCACGCCTGG

CTAATTTTTGTATTTTTAGTAGAGAGGGTTTTTCACCACGTTGGTCAGGCTGGTCTTGAACTCCTGACCTGGTGATCTGCCAGCCTCGG

CCTCCCAAAGTGCTGGAATTACAGATGTGAGCCACCATGCCCAGCCACATTTCTGTTTTGTTCAGCCATGAGATTTAATTTATTTTTGT

GATAGGAGGAACATTTATTTTTCTTTTTTTTTTTTTTCAGAAAGAGTTTCAATCTTGACATCCAGTCTGTAGTGCAACGGCAT|AAACT

AAGCTCACTGGACCCTC
```
